# Dopamine Modulates *Drosophila* Gut Physiology, Providing New Insights for Future Gastrointestinal Pharmacotherapy

**DOI:** 10.3390/biology10100983

**Published:** 2021-09-30

**Authors:** Samar El Kholy, Kai Wang, Hesham R. El-Seedi, Yahya Al Naggar

**Affiliations:** 1 Zoology Department, Faculty of Science, Tanta University, Tanta 31527, Egypt; samar_elkholy@science.tanta.edu.eg; 2 Institute of Apicultural Research, Chinese Academy of Agricultural Sciences, Beijing 100093, China; 3 Pharmacognosy Group, Biomedical Centre, Department of Pharmaceutical Biosciences, Uppsala University, Box 591, SE-751 24 Uppsala, Sweden; hesham.el-seedi@farmbio.uu.se; 4 International Research Center for Food Nutrition and Safety, Jiangsu University, Zhenjiang 212013, China; 5 Department of Chemistry, Faculty of Science, Menoufia University, Shebin El-Kom 32512, Egypt; 6 General Zoology, Institute for Biology, Martin Luther University Halle-Wittenberg, Hoher Weg 8, 06120 Halle, Germany

**Keywords:** dopamine receptors, *Drosophila*, gut motility, immunohistochemistry, Parkinson’s disease, pharmacotherapy

## Abstract

**Simple Summary:**

Dopamine is involved in a variety of physiological functions in the gastrointestinal tract (GI). Using a *Drosophila* model, we investigated the effects of dopamine administration on intestinal physiology and gut motility to gain new insights into what could be a potential future promise candidate for GI pharmacology. We investigated whether giving a dopamine-supplemented food medium to adult flies modified the gut contents, defecation rate, and excreta nature. The effects of dopamine on adult gut spontaneous contraction and motility were also studied. We discovered significant gender differences in the effect of dopamine. *Drosophila* dopamine D1-like receptors (Dop1R1 and Dop1R2) were also displayed by immunohistochemistry to be expressed in all smooth muscles in both larval and adult flies. Furthermore, we showed for the first time that dopamine drives phospholipase C Beta (PLC-β) translocation from the cytoplasm to the plasma membrane in enterocytes. Overall, the data provided new insights into the epidemiology and clinical aspects of neurodegenerative diseases associated with dopamine deficiency, as well as what may be a potential future prospect for GI pharmacotherapy.

**Abstract:**

Dopamine has a variety of physiological roles in the gastrointestinal tract (GI) through binding to *Drosophila* dopamine D1-like receptors (DARs) and/or adrenergic receptors and has been confirmed as one of the enteric neurotransmitters. To gain new insights into what could be a potential future promise for GI pharmacology, we used *Drosophila* as a model organism to investigate the effects of dopamine on intestinal physiology and gut motility. GAL4/UAS system was utilized to knock down specific dopamine receptors using specialized GAL4 driver lines targeting neurons or enterocytes cells to identify which dopamine receptor controls stomach contractions. DARs (Dop1R1 and Dop1R2) were shown by immunohistochemistry to be strongly expressed in all smooth muscles in both larval and adult flies, which could explain the inhibitory effect of dopamine on GI motility. Adult males’ gut peristalsis was significantly inhibited by knocking down dopamine receptors Dop1R1, Dop1R2, and Dop2R, but female flies’ gut peristalsis was significantly repressed by knocking down only Dop1R1 and Dop1R2. Our findings also showed that dopamine drives PLC-β translocation from the cytoplasm to the plasma membrane in enterocytes for the first time. Overall, these data revealed the role of dopamine in modulating *Drosophila* gut physiology, offering us new insights for the future gastrointestinal pharmacotherapy of neurodegenerative diseases associated with dopamine deficiency.

## 1. Introduction

The gut plays an important role in nutrient digestion and absorption, and the digestive tract’s inner lining also serves as the first line of defense against pathogens. It is also a primary source of neuronal and endocrine signals generated by the functionally important peptidergic brain–gut axis [1]. The latter has gotten a lot of research in recent years, but most of it has been on digestive functions and how brain–gut axis signals affect nutrition storage or food intake via controlling the functioning of other organs. Other studies are looking into the gut–brain axis’ other physiological functions, as well as how its dysfunction can lead to a variety of human disorders [2]. The brain–gut interactions have prompted various researchers to investigate the involvement of neuromodulators such as serotonin, norepinephrine, epinephrine, and dopamine in gastrointestinal (GI) physiology [2].

Catecholamines are physiologically active chemicals that include dopamine, norepinephrine, and epinephrine [3]. Dopamine, as a central acting catecholamine, is linked to the brain’s ‘pleasure system’, which produces emotions of pleasure and exhilaration. It also functions as a neurotransmitter, neuromodulator, and neurohormone, since it acts as an endogenous agonist for dopaminergic receptors [4]. Dopamine regulates a range of physiological and behavioral processes through the action of adenylate cyclase, which increases or decreases the cyclic adenosine monophosphate (cAMP) levels, or phospholipase C beta (PLC-β), which modulates the release of calcium in the cytosol [5,6].

In vertebrates, the food intake, digestion, and nutrient absorption through the GI tract wall are all important physiological events. Food enters the body through the GI system, and it is widely recognized that GI motility regulates feeding habits and aids in energy homeostasis regulation. Dopamine has been shown to suppress stomach motility in vitro and in vivo via binding to certain dopamine receptors (DARs) [2,7,8]; it also caused a biphasic change in the fasting motility, with an initial suppression of the stomach, followed by an increase in activity in the stomach [7]. Additionally, in the pathogenesis of Parkinson’s disease (PD), which is associated with dopamine deficiency, the enteric nervous system (ENS) symptoms such as delayed stomach emptying, constipation, and defecatory dysfunction develop early in the disease before the pathology in substantia nigra neurons [8,9,10]. As a result, it is critical to understand the role of dopamine in gut motility and, more importantly, to characterize how dopamine acts to suppress gut motility via specific DARs, as well as what is known about the involvement of the various DARs.

Dopamine has been identified as an enteric neurotransmitter and plays a variety of physiological roles in the GI tract after binding to DARs and/or adrenergic receptors [2,11]. It has its own receptors (D1 to D5) while being the precursor to epinephrine and norepinephrine. The D1-like subtypes include the dopamine D1 receptor (D1R) and D5R, whereas the D2-like subtypes include D2R, D3R, and D4R. The total GI transit time and colonic transit time are reduced in D2R knockout mice and D2R and D3R double-knockout animals but not in D3R knockout mice, suggesting that endogenous dopamine inhibits intestinal motility predominantly through D2R activation [12]. Furthermore, D2R and D3R have been demonstrated to play a function in the regulation of stomach emptying in rats in several investigations [13,14]. The subcutaneous administration of the D3R agonists S (+)-PD 128,907 and R (+)-7-OH-DPAT, as well as the D2R agonist quinpirole, for example, delayed stomach emptying in rats in a dose-dependent manner. Neither the selective D1R and D5R agonist SKF38393 nor the selective D4R agonist PD 168,077, on the other hand, delayed stomach emptying in rats. As a result, a deeper understanding of the various roles of DARs in gut motility has great potential for gastrointestinal pharmacology development.

Signal transmission in gastric and intestinal smooth muscle is facilitated by receptors that are linked to various effector enzymes via different G proteins, such as phosphoinositide-specific phospholipase C (PLC-1 and PLC-3) and phosphatidylcholine (PC)-specific PLC, PLD, and PLA2. These enzymes are activated differently in circular and longitudinal muscle cells, resulting in Ca^2+^ mobilizing (IP3, AA, and cADPR) and other (DAG) messengers responsible for the initial and sustained phases of gut contraction, respectively [15]. According to various studies, dopamine and D1-like receptor agonists can trigger PLC-mediated phosphoinositide hydrolysis in native mammalian brain tissues (reviewed in Reference [16]). However, it is yet to be determined whether dopamine, as an endogenous ligand of dopaminergic receptors, might mediate gut peristalsis by activating PLC activity in gut cells.

In *Drosophila*, ~60 percent of the genes linked to human disorders have a *Drosophila* counterpart. The intestines of humans and flies have similar tissue, morphology, and physiological functions [17,18]. Furthermore, the availability of genetic tools makes *Drosophila melanogaster* a good model. One of these tools is the GAL4/UAS system, which consists of two parts: the GAL4 gene, which encodes the yeast transcription activator protein Gal4, and the UAS (Upstream Activation Sequence), an enhancer to which GAL4 specifically binds to activate gene transcription, allowing researchers to study gene functions both temporally and spatially [19]. In this study, based on a *Drosophila* model, we investigated the effects of dopamine on the intestinal physiology and gut motility to gain new insights into what could be a potential future promise for GI pharmacology. Our main question is whether feeding *Drosophila* flies food medium supplemented with dopamine will modulate the gut content, defecation rate, the nature of excreta, and gut motility. We also employed the GAL4/UAS system to knock down particular dopamine receptors in various neurons or enterocytes cells using specific GAL4 driver lines that help us to determine which dopamine receptor plays a primary role in controlling gut contractions.

## 2. Materials and Methods

### 2.1. Fly strains and Culture

The following *Drosophila melanogaster* lines were purchased from the Bloomington Drosophila Stock Center (Dept of Biology, Indiana University, Bloomington, IN, USA) and utilized in all the experiments: w1118, Dop1R1-RNAi (#31765), Dop1R2-RNAi (#26018), and Dop2R-RNAi (#26001) and GAL4 driver lines: Dop1R1-GAL4 (#39609), Dop1R2-GAL4 (#46640), Dop2R-GAL4(#49416), NP1-GAL4 (#84307), nSyb-GAL4 (#51941), and UAS-GFP (#5428). The flies were maintained on standard *Drosophila* food media comprising cornmeal agar (14 to 15-g agar, 18.5-g yeast, 61-g glucose, 30.5-g sucrose, and 101-g corn meal/L, then kept at 25 °C and 50–60% relative humidity (RH) with an 18/6-h light/dark cycle) [20,21,22].

### 2.2. Chemicals

Dopamine (Sigma-Aldrich, catalog# H8502-5G, Schnelldorf, Germany) was dissolved in ddH2O to obtain a stock solution with a concentration of 10 M, and the desired working solutions were then prepared. The primary antibody was anti-GFP (green fluorescent protein) rat monoclonal IgG from Santa Cruz Biotechnology (Heidelberg, Germany), and the secondary antibody was donkey anti-Rat IgG from Jackson Immuno Research Europe (Heidelberg, Germany).

### 2.3. Gut Contents

To test whether feeding adult flies (male and female) food media supplemented with dopamine would increase the gut contents or not, we quantified the gut content according to Reference [23]. Briefly, groups of 2-h-starved males and females (*n* = 5) were fed for 24 h on blue media spiked with 300 μL of dopamine concentration ranging from 0.001 to 1 M on top of the normal fly food. The food vials were kept at 25 °C at least 6–8 h until the dopamine was completely absorbed through the media. For the control food (without dopamine), ddH_2_O alone was added. The guts of three individuals were then dissected in distilled water and centrifuged; the first supernatant was discarded, then centrifuged again, and the blue dye in the second supernatant was measured at 590 nm using a nanodrop ND-1000 spectrophotometer. The measurements were repeated six times. Adults fed on blue media without dopamine were used as the control.

### 2.4. Area, Perimeter, Integrated Dye Intensity, and Number of Fecal Deposits

The fecal deposits were counted on the clear walls of the dopamine-supplemented blue media [23]. To do that, groups of males and females (*n* = 5) were starved for 2 h prior to the onset of the experiment. Each group was fed for 24 h on blue media supplied with a dopamine concentration ranging from 10^−5^ to 10^−3^ M in a normal food vial or in a Petri dish. All 24-h fecal deposits at a distance of 4 cm from the surface of the food in the vial or in the Petri dishes lids were recorded. The experiments were done in 6 replicas. Using ImageJ, the fecal pellets area, perimeter, and integrated dye intensity were calculated. The integrated dye intensity was calculated as the product of the Area and Mean Gray Value (average gray value within each deposit), which were calculated by converting each pixel to grayscale using the formula: gray = 0.299 red + 0.587 green + 0.114 blue. The results were expressed as the mean ± S.E.M.

### 2.5. Fecal Pellets (pH) Hue Analysis

To test whether dopamine affects the nature of *Drosophila* excreta, we use phenol red as the pH indicator to get colored fecal deposits [23]. Six groups of adult males or females fed for 24 h on normal food media supplied with dopamine concentrations ranged from 10^−5^ to 10^−3^ M and phenol red (0.5 M in 10% glucose) as a nonabsorbable pH marker in 50-mm Petri dishes. After 24 h, digital images of Petri dish lids were obtained using an Olympus BX61 light microscope. The mean red–green–blue values obtained from ImageJ were converted to hue–saturation–brightness and the corresponding color patches using a free RGB-to-HSB tool. The H value (hue) is computed as the difference between the two lowest values divided by the maximum value and expressed in degrees (0–360). Hue values refer to the pH of the fecal pellets. Patch colors are based on hexadecimal HTML codes. The hue histograms were obtained using ImageJ software with color mode HSB.

### 2.6. Gut Peristalsis

The gut contraction assay was used to assess the ex vivo effect of dopamine on adult gut spontaneous contraction and motility [24]. Briefly, an individual cold-anesthetized adult *D. melanogaster* was pinned dorsal-side down onto a dissecting Petri dish, and the animal was covered with physiological saline solution (5-mM HEPES, 128-mM NaCl, 36-mM sucrose, 4-mM MgCl2, 2-mM KCl, and 1.8-mM CaCl2, pH 7.1). The cuticle at the thorax–abdomen junction was removed, revealing the underlying gut tissue. Then, the contractions were recorded for 30 s at the base of the crop duct, followed by a 30-s pause. This paradigm was repeated five times in a 5-min period to measure the basal contraction rate. The saline was then removed and replaced with either saline (control) or saline containing 10^−3^-M dopamine, and the number of contractions was counted for 30 s followed by a 30-s interval, repeating this paradigm ten times over a 10-min period to determine the experimental contraction rate. This procedure was carried out five times (5 males and 5 females). The in vivo effect of dopamine on spontaneous contractions of the larval midgut was studied using a modified version of Reference [24]. To avoid injury to the CNS and stomach, an individual larva was placed dorsal-side up on double-sided sticky tape, and a small cut was formed lateral to the midline with fine scissors. The physiological saline solution or the 10^−3^-M dopamine-containing saline was administered to the top immediately, and the contractions were monitored for 30 s followed by a 30-s pause, repeating this paradigm ten times.

We then investigated whether any possible dopamine-induced responses in gut motility are mediated by various neurons, including those innervating the gut or through other pathways, such as gut enterocytes cells, using the loss-of-function (RNAi knockdown) of the corresponding dopamine receptors. Specific GAL4 driver lines either directed in various neurons (nsyb driver line) or in enterocytes cells (NP1 driver line) were used. The guts of F1 individuals were dissected in saline; then, the gut contractions were calculated as mentioned above. F1 of the GAL4 driver lines crossed with W^1118^ were used as the control for the RNAi-mediated gene silencing experiments.

### 2.7. Activation of Phospholipase C Beta (PLC-β)

To determine whether dopamine, an endogenous ligand of dopaminergic receptors, stimulates PLC activity, we crossed UAS-PLC-mRFP with NP1-GAL4 and performed an ex vivo assay in the gut of F1 adult flies. The adult flies (*n* = 10) had their stomachs dissected in a hemolymph-like (HL3) buffer. Dopamine (10–3 M in HL3) was given to the top of the tissue immediately after dissection, and the translocation of PLC-β particles was observed using a SP5 Leica confocal microscope. In this experiment, the guts of F1 given a HL3 buffer without dopamine were employed as the control.

### 2.8. Expression Pattern of Dopaminergic Receptors in the Guts of Larvae and Adults

Dopaminergic receptor expression patterns in the guts of F1 were investigated using immunohistochemistry after crossing the Dop1R1, Dop1R2, and Dop2R GAL4 driver lines with the UAS GFP reporter lines. The dissected gut preparations were rinsed twice with PBS, fixed with 4 percent of PFA in PBS overnight at 4 °C, washed three times with PBST, and then blocked for 30 min at room temperature in a blocking buffer (1XPBS + 2% Triton + 10% goat serum). The primary antibody (anti-GFP rat monoclonal IgG; 1:200 in a blocking buffer) was incubated overnight at 4 °C, followed by three 5-min washes in PBST (0.1 percent Triton-100). The secondary antibody (donkey anti-Rat IgG; 1:500 in a blocking buffer) was incubated for 1–3 h at room temperature with TRITC-labeled Phalloidin (0.5 g/mL). The tissues were then washed three times in PBS-T for five minutes each time. Glycerol was used as a mounting medium for the preparations, which were then analyzed using a Leica SP5 confocal microscope.

### 2.9. Statistical Analysis

To analyze the data, we utilized GraphPad Prism v. 8.0 for Windows. The data were changed to log10 when necessary to better estimate the variance’s normality and homogeneity. To investigate the differences in the gut content, number of fecal pellets, perimeter, and integrated dye intensity, a one-way analysis of variance (ANOVA), followed by Tukey’s Multiple Comparison test, were performed. The Student’s *t*-test was performed to assess the gastrointestinal motility in larvae and adult flies following dopamine treatment to the control. A liner regression analysis was used to see if there was a link between the gut content and the number of fecal pellets in relation to varied dopamine concentrations. The Pearson correlations coefficient was also used to assess the relationship between the effect of dopamine on the gut contents and fecal pellet counts in male and female flies to determine if the change in the gut contents is due to a change in the defecation or food consumption rates. The differences were considered significant at *p* < 0.05.

## 3. Results

### 3.1. Effect of Dopamine on the Gut Contents

**Figure 1:**
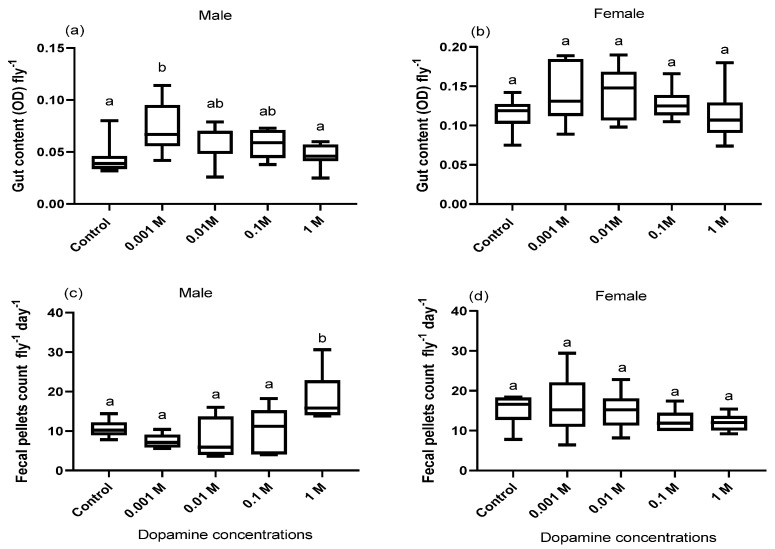
The gut content (**a**,**b**) and the fecal pellets count (**c**,**d**) of adult flies (*n* = 5) fed on blue food media spiked with different concentrations of dopamine compared to control flies fed standard food media without dopamine. Symbols on the box plot represent the maximum and minimum values (whiskers: ┬ ┴) and mean values (-). Different lowercase letters indicate statistically significant differences between treatments (one-way ANOVA with Tukey’s post hoc test, *p*  <  0.05).

Feeding adult flies (males and females) media supplemented with dopamine increased the gut contents of only males at the least conc. of dopamine (0.001 M) as compared to control flies fed on normal food media without dopamine (F = 5.06, df = 4, *p* = 0.002; Figure 1a). Linear regression, however, revealed that the gut contents were significantly and negatively correlated with the increase in dopamine conc. for both male (R^2^ = 0.26, *p* = 0.01; Figure S1a) and female flies (R^2^ = 0.14, *p* = 0.03; Figure S1b).

### 3.2. Effect of Dopamine on Defecation Rate

Fecal deposits were counted on the clear walls of dopamine-supplemented blue media to quantify the effect of dopamine on the defecation rate of both male and female flies. Sex-specific discrepancy was observed where only male flies fed media spiked with the highest concentration of dopamine (1 M) had substantially more fecal pellets than the control male flies fed standard food media without dopamine (F = 5.21 df = 4, *p* = 0.003; Figure 1c). In addition, linear regression revealed that, in only the males, the number of fecal pellets was significantly associated with the increase in dopamine concentration (R^2^ = 0.38, *p* = 0.001; Figure S1c), while the females did not show these alterations.

We also evaluated the relationship between the effect of dopamine on the gut contents and fecal pellet counts in male and female flies to find out whether the change in gut contents is due to a change in the defecation or food consumption rates. The change in the gut contents and fecal pellets showed a nonsignificant correlation (males: *r* = 0.49, *p* = 0.39; females: *r* = 0.88, *p* = 0.11; Figure S1e,f), indicating a potential role for dopamine in modulating the feeding behavior.

**Figure 2:**
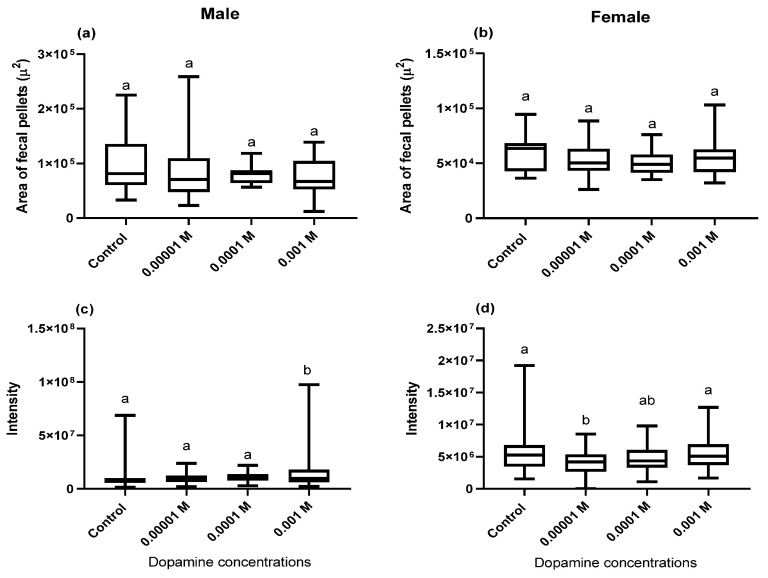
The area of fecal pellets (µ) (**a,b**) and dye intensity in the fecal pellets (**c**,**d**) of adult male and female flies (*n* = 5) fed on blue food media spiked with different concentrations of dopamine compared to control flies fed standard food media without dopamine. Symbols on the box plot represent the maximum and minimum values (whiskers: ┬ ┴) and mean values (-). Different lowercase letters indicate statistically significant differences between treatments (one-way ANOVA with Tukey’s post hoc test *p*  <  0.05).

There was no significant change in the perimeters of the fecal pellets of both males and females maintained in dopamine-supplemented food media in comparison to the control flies fed on regular food media without dopamine (males: F = 1.33, df = 3 *p* = 0.26; females: F = 1.56, df = 3, *p* = 0.66; Figure 2a,b). The integrated intensity of the blue dye in the fecal pellets of both male and female flies fed dopamine-supplemented food media also changed significantly compared to the control flies and was conc.-dependent (males: F = 15.56, df = 3, *p* = 0.001; females: F = 6.60, df = 3, *p* = 0.001, Figure 2c,d).

### 3.3. Fecal Pellets (pH) Hue Analysis

According to the fecal pellets hue analysis shown in (Figure S2), dopamine induced the acidification of the female flies’ deposits but had no effect on the pH of the male flies’ deposits.

### 3.4. Gut Peristalsis

**Figure 3:**
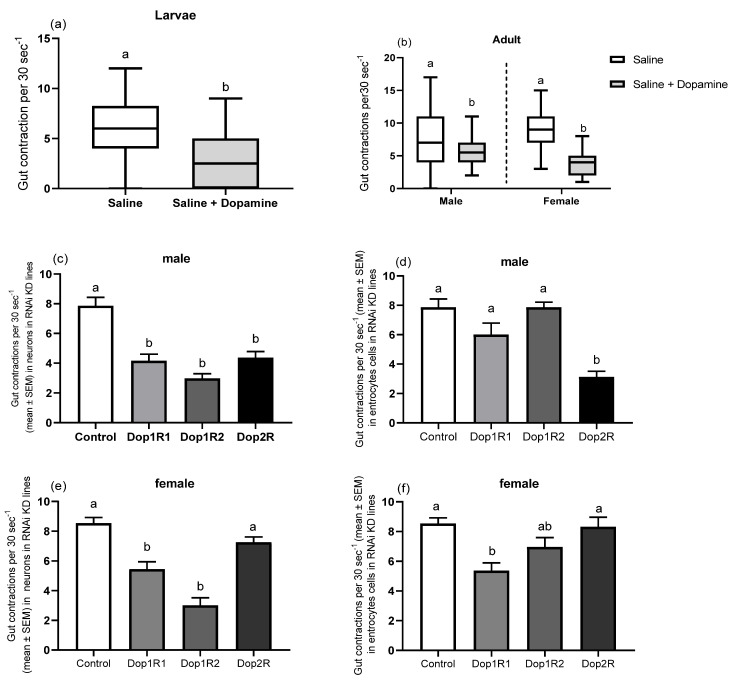
Gut motility assay (number of gut contractions per 30 s) of (**a**) larvae and (**b**) adult flies (males and females, *n* = 5). Symbols on the box plot represent the maximum and minimum values of the gut contractions per 30 s (whiskers: ┬ ┴) and mean values (-). Different lowercase letters indicate significant differences among treatments (Student’s *t*-test, *p * <  0.05). (**c**,**d**) Gut motility in male flies and (**e**,**f**) gut motility in female flies (mean  ±  SEM) that lost the function of dopamine receptors (RNAi knockdown) using specific GAL4 driver lines either directed in various neurons, including those in the gut (nsyb driver line) (**c**,**e**) or in enterocyte cells (NP1 driver line) (**d**,**f**). F1 of the GAL4 driver lines crossed with W^1118^ were used as the control for the RNAi-mediated gene silencing experiments. Column bars with different lowercase letters denote significant differences among treatments (one-way ANOVA with Tukey’s post hoc test, *p*  <  0.05).

Dopamine significantly decreased the frequency of the spontaneous contraction of the gut of both larvae and adult flies (males and females) (larvae: t = 4.37, df = 88, *p* < 0.001; males: t = 2.99, df = 89, *p* = 0.003; females: t = 10.19, df = 99, *p* < 0.001; Figure 3a,b). To elucidate the underlying mechanisms of the dopamine-induced responses in gut motility, the RNAi-mediated silencing of specific receptors in various neurons, including those in the gut or in gut enterocytes, was carried using the nsyb and NP1 driver lines, respectively. RNAi effector and GAL4 driver lines crossed with W^1118^ were used as the controls. We observed sex-specific differences in dopamine receptor-defective adults, where adult males’ gut peristalsis was significantly suppressed by knocking down dopamine receptors Dop1R1, Dop1R2, and Dop2R of different neurons (F = 20.83, df = 3, *p* < 0.001; Figure 3c) and only targeting the Dop2R silencing of gut EECs compared to the control (F = 16.50, df = 3, p < 0.001; Figure 3d). We found that targeting the loss-of-function of both Dop1R1 and Dop1R2 of different neurons (F = 25.28, df = 3, *p* < 0.001; Figure 3e) and only targeting Dop1R1 silencing of enterocytes cells of the gut significantly induced gut motility suppression in adult female flies compared to the control (F = 7.52, df = 3, *p* < 0.001; Figure 3c,f). However, knocking down the Dop2R dopamine receptor either in different neurons or in gut enterocyte cells did not affect the gut motility, indicating that Dop2R receptor activity has little effect on the gut motility of female flies (Figure 3e,f).

### 3.5. Expression Pattern of Dopaminergic Receptors

**Figure 4:**
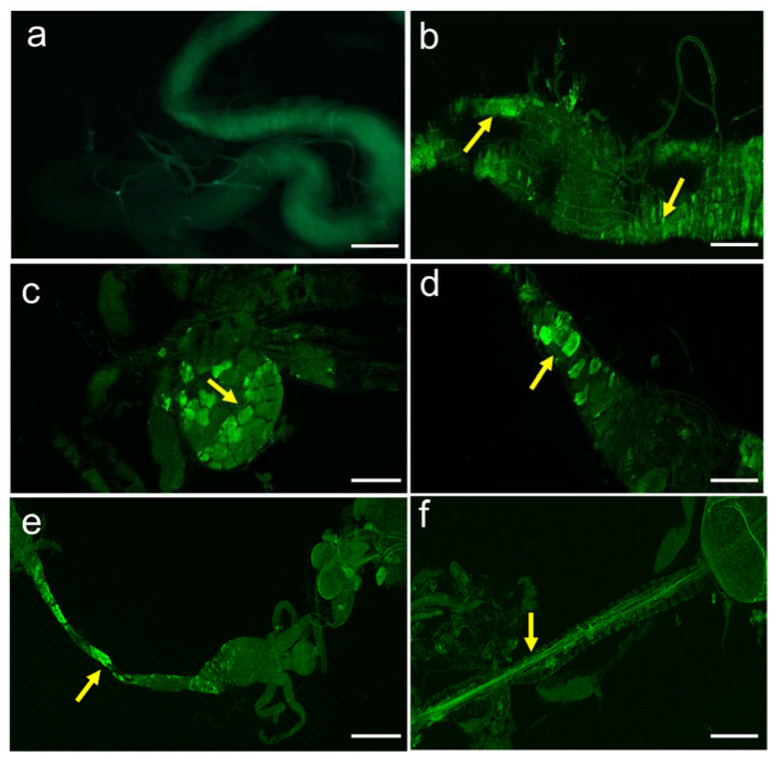
Expression pattern of dopamine receptor Dop1R1 in the nerves and enterocytes of *Drosophila* larvae stained immunohistochemically using the anti-GFP antibody. (**a**) Negative control (i.e., the midgut of larvae that did not show up the expression of Dop2R). The arrows in (**b**) refer to the expression of Dop1R1 in the midgut cells, (**c**) in proventriculus, and (**d**) in the foregut cells. Dop1R1 expressed in (**e**) foregut cells, and (f) nerves innervating the foregut. Scale bars: (**b**,**d**) 50 µm, (**a**,**c**,**f**) 100 µm, and (**e**) 500 µm.

**Figure 5:**
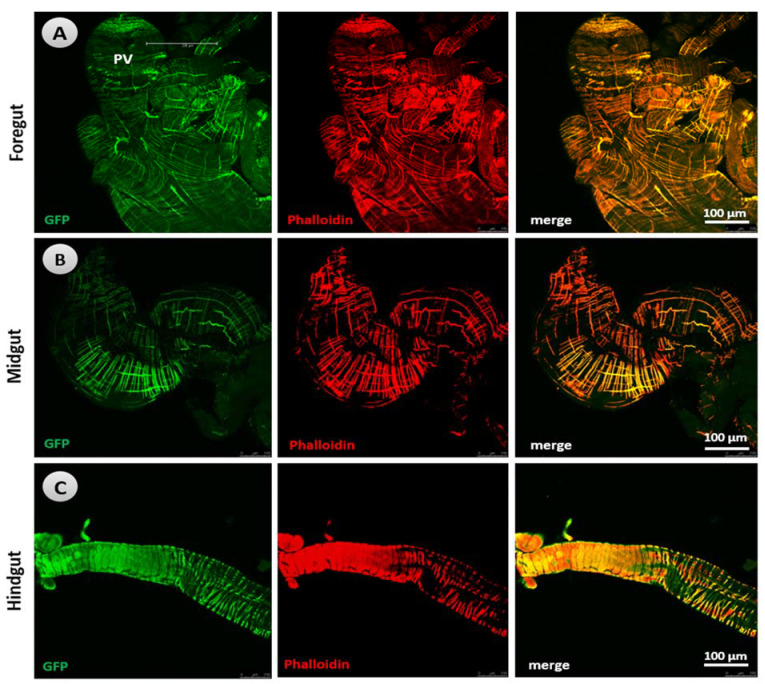
Dop1R1 expression pattern in the larval gut musculature stained immunohistochemically using the anti-GFP antibody (green florescent micrograph) with phalloidin (red florescent micrograph), and the 365 overlay of these two florescent micrographs revealed that that almost all muscles express the Dop1R1 receptor: (**A**) foregut, (**B**) midgut, and (**C**) hindgut. PV: Proventriculus.

**Figure 6:**
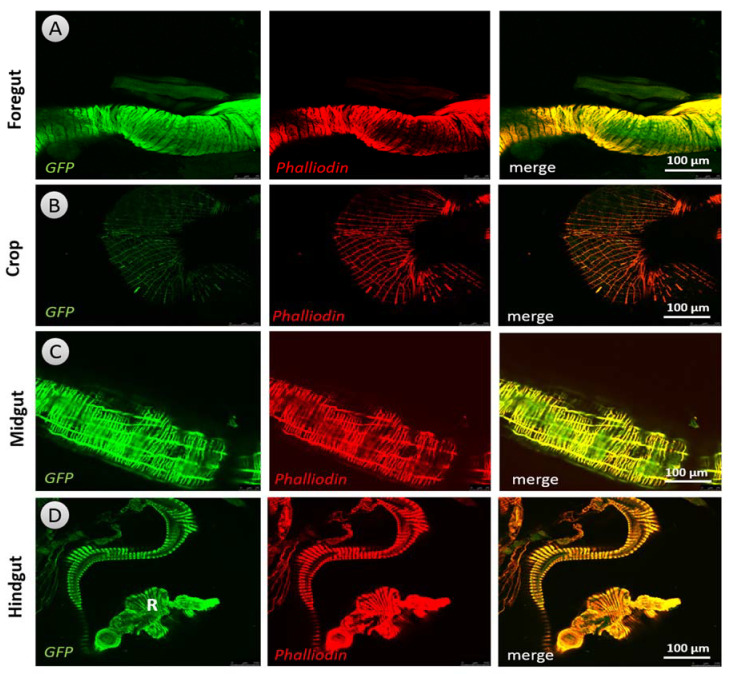
Dop1R1 expression pattern in adult flies’ (male and female) gut musculature stained immunohistochemically using anti-GFP antibody (green florescent micrograph) with phalloidin (red florescent micrograph), and the overlay of these two florescent micrographs revealed that almost all muscles express the Dop1R1 receptor: (**A**) foregut, (**B**) crop, (**C**) midgut, and (**D**) hindgut. R: rectum.

DARs have been identified in nerves and gut tissues (enteroendocrine and enterocytes) of both larvae (Figure 4 and Figure S3) and adult *Drosophila* (Figure S4) using the anti-GFP antibody. As a negative control for all DAR expression patterns, the midgut of larvae that did not display an expression of Dop2R were employed. Our immunohistochemistry results revealed that Dop1R1 (Figure 5 and Figure 6) and Dop1R2 (Figures S5 and S6) are strongly expressed throughout larval and adult gut musculature (foregut, midgut, and hindgut). A weak Dop2R expression was also found however, in only adult hindgut (data not shown). The expression pattern was determined by crossing the GAL4 with UAS-GFP. The localization of Dop1R1 fluorescence in the gut musculature of both larval and adult flies was also quantified using ImageJ software, which revealed that Dop1R1 expression varied in different gut tissues (Figures S7 and S8).

### 3.6. Phospholipase C Beta (PLC-β)

**Figure 7:**
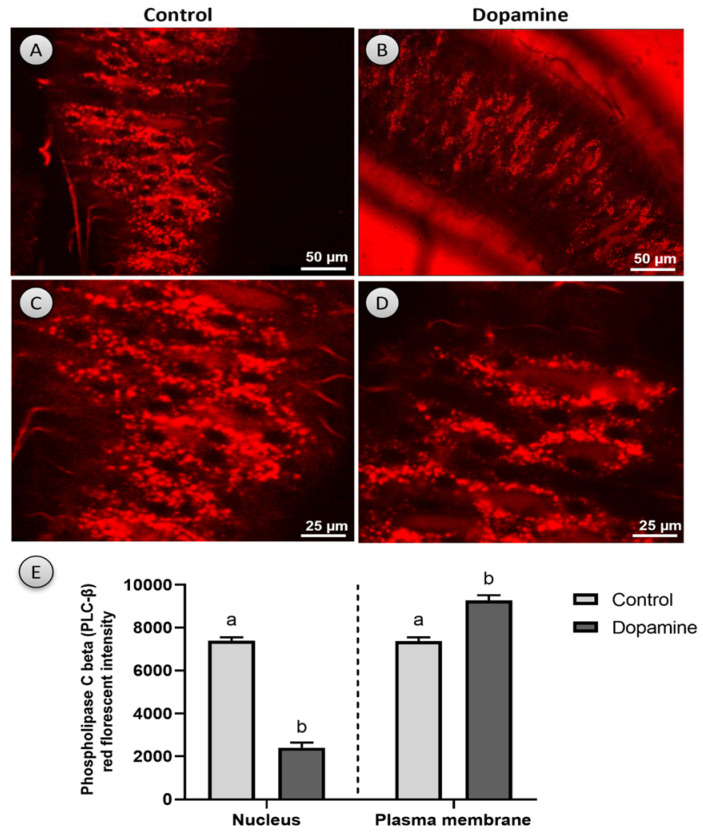
Phospholipase C beta (PLC-β) translocation in enterocytes cells of the gut of F1 flies (*n* = 10) results from the crossing of UAS-PLC-mRFP with NP1-GAL4 in the control (**A**,**C**) and after dopamine 10^−3^ M application. (**B**,**D**,**E**) Quantification of PLC-red fluorescence localization in the nucleus and cell membrane in the control and after dopamine application; dopamine activates the translocation of PLC- from the cytoplasm to the plasma membrane in enterocyte stem cells more quickly than a control. Column bars with different lowercase letters denote significant differences among treatments (Student’s *t*-test, *p*  <  0.05).

Our ex vivo findings revealed that dopamine (10^−3^ M) rapidly activates the translocation of PLC-β from the cytoplasm to the plasma membrane and, as a result, is expected to enhance the intracellular Ca^2**+**^ mobilization in enterocytes as opposed to the control, in which PLC-β remains in the cytoplasm (Figure 7A–C and Supplementary Materials Video S1). After around 20–25 min post the dopamine administration, the initial distribution of PLC-β was restored. The localization of PLC-red fluorescence in the nucleus and cell membrane in the control and after dopamine application was also quantified using ImageJ software. When dopamine was applied, the intensity of PLC-red fluorescence in the nucleus reduced dramatically, whereas it increased significantly in the cell membrane (nucleus: t = 17.02, df = 4, *p* *<* 0.001; plasma membrane: t = 6.37, df = 4, *p* < 0.01; Figure 7E).

## 4. Discussion

Dopamine has been identified as an enteric neurotransmitter and is involved in a number of physiological functions in the GI tract [2,11]. Szabo’s early work (1985) confirmed the connection between the brain dopamine and the peripheral gastric disease. We discovered that dopamine plays a role in *Drosophila* gut physiology modulation through a series of experiments, which could open the way for future GI pharmacotherapy.

Neuropeptides that regulate the energy balance (homeostatic processes) via the hypothalamus are known to modulate the activity of dopamine cells and their projections into regions involved in the rewarding processes behind food intake [25]. Only male flies fed food media enriched with dopamine demonstrated an increase in gut contents at the lowest concentration of dopamine (0.001 M) evaluated in this study. However, for both male and female flies, linear regression revealed that the gut contents were significantly and negatively associated with increased dopamine levels in food. These findings are in line with those of Reference [26], who found that excessive dopamine signaling inhibits eating in mice. Since dopamine is involved in the development of food preferences, dietary alterations with or without compulsive eating could be a sign of a change in appetite caused by excessive dopaminergic neurotransmission. However, the dopaminergic system’s role in feeding behavior is complicated and poorly understood. It appears to have various effects on different circuits and release patterns (phasic versus chronic release) [26,27]. More research is therefore required to determine the role of dopamine and DARs in modulating feeding behavior.

Chronic constipation is the most common symptom, affecting up to 89% of PD patients [28]. Levodopa (L-DOPA), which is used for treatment for PD motor symptoms [29], works by being converted to dopamine; however, this medication can also cause diarrhea or worse constipation by altering dopaminergic signaling [30]. In the current investigation, only male flies fed food media spiked with the highest dose of dopamine (1 M) had considerably more fecal pellets than the control flies. In addition, linear regression demonstrated that the number of fecal pellets was substantially related with an increase in the dopamine concentration in only the males, whereas the females showed no such changes. On the other hand, dopamine increased the acidity of the female flies’ deposits but had no effect on the pH of the male flies’ deposits. These gender differences could provide new insights into the epidemiology and clinical characteristics of PD. Since PD patients have a distinctive clinical profile, depending on their gender, men are twice as likely as women to develop it; however, women had a greater mortality rate and a faster illness progression [31,32,33,34].

In vitro and in vivo studies have demonstrated that dopamine inhibits stomach motility via binding to specific DARs [2,7,8]. Similarly, dopamine inhibited the gut motility in both larval and adult flies in the present study. Furthermore, knocking down DARs (Dop1R1 and Dop1R2) in both male and female flies showed a decrease in gut motility. Moreover, knocking down Dop2R, which is weakly expressed in both male and female hindgut cells, reduced the gut motility in only the male flies, a finding that is consistent with previous studies in mammals, which found a reduction in the total GI transit time and colonic transit time in D2R knockdown mice and D2R and D3R double-knockout animals but not in D3R knockdown mice, implying that endogenous dopamine inhibits intestinal motility primarily through the D2R receptor [14]. These findings are very similar to the effects of PD on intestinal motility, in which a lack of dopamine causes constipation [35]. However, it is unknown if both dopamine feeding and receptor knockdown can inhibit stomach contractions. If that is the case, which receptor is dopamine acting on to cause suppression in this situation? More research is therefore needed to look at this point and its pharmacological implications.

Although biological sex variations in the nigrostriatal dopaminergic (NSDA) system, which are regulated by hormonal, genetic, and environmental variables, are mostly responsible for sex differences in PD [34], knowing the involvement of DARs in these sex differences is fascinating. Our data show consistent gender differences, with dopamine affecting the gut content and fecal pellet count in male flies, as well as Dop2R knockdown reducing the gut motility in exclusively male flies. Similarly, References [36,37] discovered that gene expression profiles in normal substantia nigra pars compacta (SNc) DAergic neurons are sex-specific, suggesting a male bias that may contribute to PD vulnerability. Furthermore, different mechanisms are involved in adaptation processes in the male and female surviving DAergic neurons, implying that the nature of the disease, and maybe the response to treatment, is sex-specific. As a result, a study into the role of DARs in sex variations in the presentation, progression, and treatment responses in PD could be extremely beneficial in terms of improving the clinical diagnosis and therapy.

Similarly, cocaine-induced dopamine increases lowered DAR signaling considerably, changing the balance between D1R and D2R signaling [38], affecting the gut physiology, homeostasis [39], and microbiota [40]. On the other hand, domperidone, a specific D2 receptor antagonist, has been shown to improve myogenically transmitted antroduodenal coordination in guinea pigs [41], and 5-HT4 receptor agonists such as mosapride and tegaserod have been shown to promote stomach emptying in people with PD, possibly by increasing the local Ach release [42]. In addition, DARs (Dop1R1 and Dop1R2) were discovered to be expressed in all smooth muscles, as well as in nerves and gut tissues (enteroendocrine and enterocytes) of both larvae and adult flies in this study. Additionally, in the proximodistal axis of the mice gut, Reference [12] discovered transcripts encoding all five types of DARs, as well as immunoreactivities of all of these receptors, except D4, in the layers of the colon-containing neurons. As a result, it is probable that dopamine influences the musculature to regulate intestinal physiology locally.

PLCs are ubiquitous enzymes that allow cells to communicate with their surroundings by transducing signals from the heterotrimeric G proteins of the Gq family [43,44,45]. Phosphatidylinositol-4,5-bisphosphate (PIP2) is hydrolyzed by PLC via a receptor, resulting in the release of second messengers diacylglycerol (DG) and inositol-1,4,5-triphosphate (IP3) [46,47]. IP3 raises cytosolic calcium by encouraging its release from intracellular storage locations such as the endoplasmic reticulum, whereas DG is a protein kinase C (PKC) stimulator [46,47]. PLC-mediated phosphoinositide hydrolysis can be triggered by dopamine and D1-like receptor agonists, however, in native mammalian brain tissues (reviewed in Reference [5]). Our findings, on the other hand, showed for the first time in vivo that dopamine rapidly promotes the translocation of PLC- from the cytoplasm to the plasma membrane, and as a result, it is expected to enhance the intracellular Ca^2+^ mobilization in *Drosophila* enterocytes that may activate the subsequent signal pathways, including those involved in the inhibition of gut contractions. In the same context, intracellular calcium increases have been shown to play a role in the initial and persistent phases of gut contraction (reviewed in Reference [17]) and on the activity and/or structure of numerous cellular proteins [48]. Furthermore, a PLC/IP3 pathway has been shown to be involved in selectively regulating dopamine-mediated locomotor activity in mice [16]. As a result, the current findings underscore the critical role of dopamine in the control of *Drosophila* gut physiology, potentially opening the way for future GI pharmacotherapy.

## 5. Conclusions

Taken together, we discovered significant gender differences in effects of dopamine on the gut content, defecation rate, excreta pH in *Drosophila* flies, and in dopamine receptor-defective adults, which provide new insights into the epidemiology and clinical aspects of PD. Immunohistochemical investigations revealed the existence of dopaminergic receptors in gut muscles, while functional studies demonstrated dopamine effects on the gut motility, elucidating dopamine’s role in the gut physiology. Our study also indicated that dopamine induces PLC to translocate from the cytoplasm to the plasma membrane for the first time in enterocytes, potentially activating downstream signal pathways, such as those involved in gut contraction inhibition. Overall, these data provided new information on what could be a potential future prospect for GI pharmacotherapy patients and PD patients.

## Data Availability

All data are presented and available in the manuscript. Additional information regarding the manuscript will be welcome by the authors.

## Supplementary Materials

The following are available online at https://www.mdpi.com/article/10.3390/biology10100983/s1. Figure S1. Relationships between different concentrations of dopamine, the gut content (a,b), and number of fecal pellets (c,d) excreted in male and female *Drosophila* flies fed on blue food media spiked with different concentrations of dopamine. Figure S2. Hue, pH, and patch color analysis of (I) male and (II) female (*n* = 6) fecal pellets after being fed on normal food media supplemented with different concentrations of dopamine. Figure S3. GFP immunoreactivity in the Dop1R1 GAL4::UAS GFP larval gut tissues. Figure S4. Dopamine receptors (DARs) in the nerves and gut tissues of adult *Drosophila* stained immunohistochemically using the anti-GFP antibody. Figure S5. Dop1R2 expression pattern in the larval gut musculature. Figure S6. Dop1R2 expression pattern in an adult fly’s gut musculature. Figure S7. Quantification of Dop1R1 fluorescence localization in the larval gut musculature. Figure S8. Quantification of Dop1R1 fluorescence localization in both larval and adult gut musculatures stained immunohistochemically using anti-GFP antibody with phalloidin (red florescent micrograph).
