# Supplementary material for: Dopamine Modulates Drosophila Gut Physiology, Providing New Insights for Future Gastrointestinal Pharmacotherapy
Source: Biology (Basel). 2021 Sep 30;10(10):983. doi: 10.3390/biology10100983 (PMC8533061; doi:10.3390/biology10100983)
Supplement: Supplementary file 1 [file biology-10-00983-s001.zip › biology-1307163-supplementary.pdf]

biology

Article

# Dopamine Modulates *Drosophila* Gut Physiology, Providing New Insights for Future Gastrointestinal Pharmacotherapy

**Samar El Kholy [1], Kai Wang [2,*], Hesham R. El-Seedi [3,4,5], and Yahya Al Naggar [1,6,*]**

[1] Zoology Department, Faculty of Science, Tanta University, Tanta 31527, Egypt; Samar_elkholy@science.tanta.edu.eg
[2] Institute of Apicultural Research, Chinese Academy of Agricultural Sciences, Beijing 100093, China
[3] Pharmacognosy Group, Biomedical Centre, Department of Pharmaceutical Biosciences, Uppsala University, Box 591, SE-751 24 Uppsala, Sweden; hesham.el-seedi@farmbio.uu.se
[4] International Research Center for Food Nutrition and Safety, Jiangsu University, Zhenjiang 212013, China
[5] Department of Chemistry, Faculty of Science, Menoufia University, Shebin El-Kom 32512, Egypt
[6] General Zoology, Institute for Biology, Martin Luther University Halle-Wittenberg, Hoher Weg 8, 06120 Halle, Germany
* Correspondence: kaiwang628@gmail.com (K.W.); yehia.elnagar@science.tanta.edu.eg (Y.A.N.); Tel.: +86-10-62593411 (K.W.); +49-345-55-26503 (Y.A.N.)

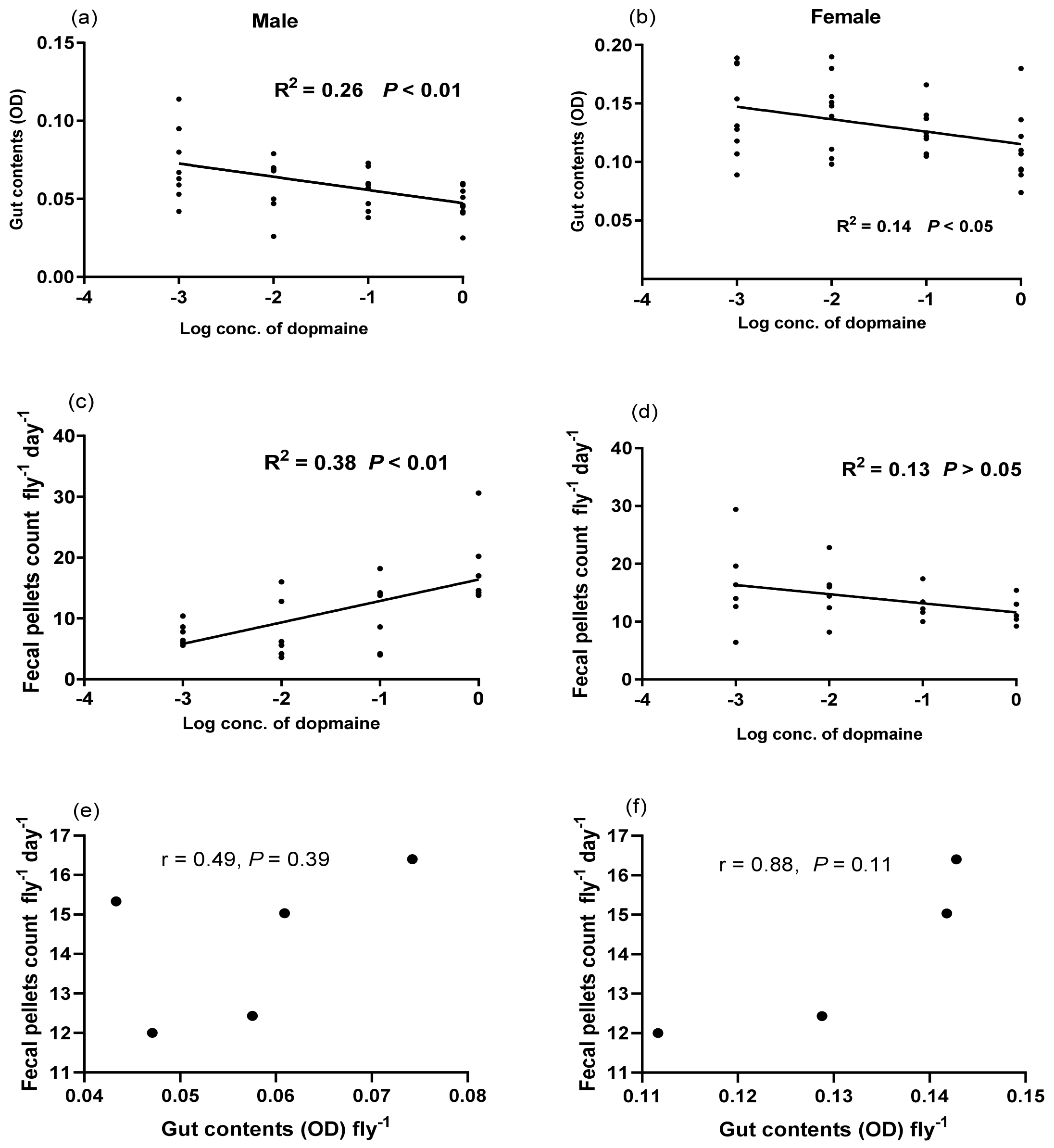


**Figure S1.** Relationships between different concentrations of dopamine, gut content (**a**,**b**) and number of fecal pellets (**c**,**d**) excreted in male and female *Drosophila* flies fed on blue food media spiked with different concentrations of dopamine. (**e**,**f**) Pearson *r* correlations between gut contents and fecal pellets count in both male and female flies.

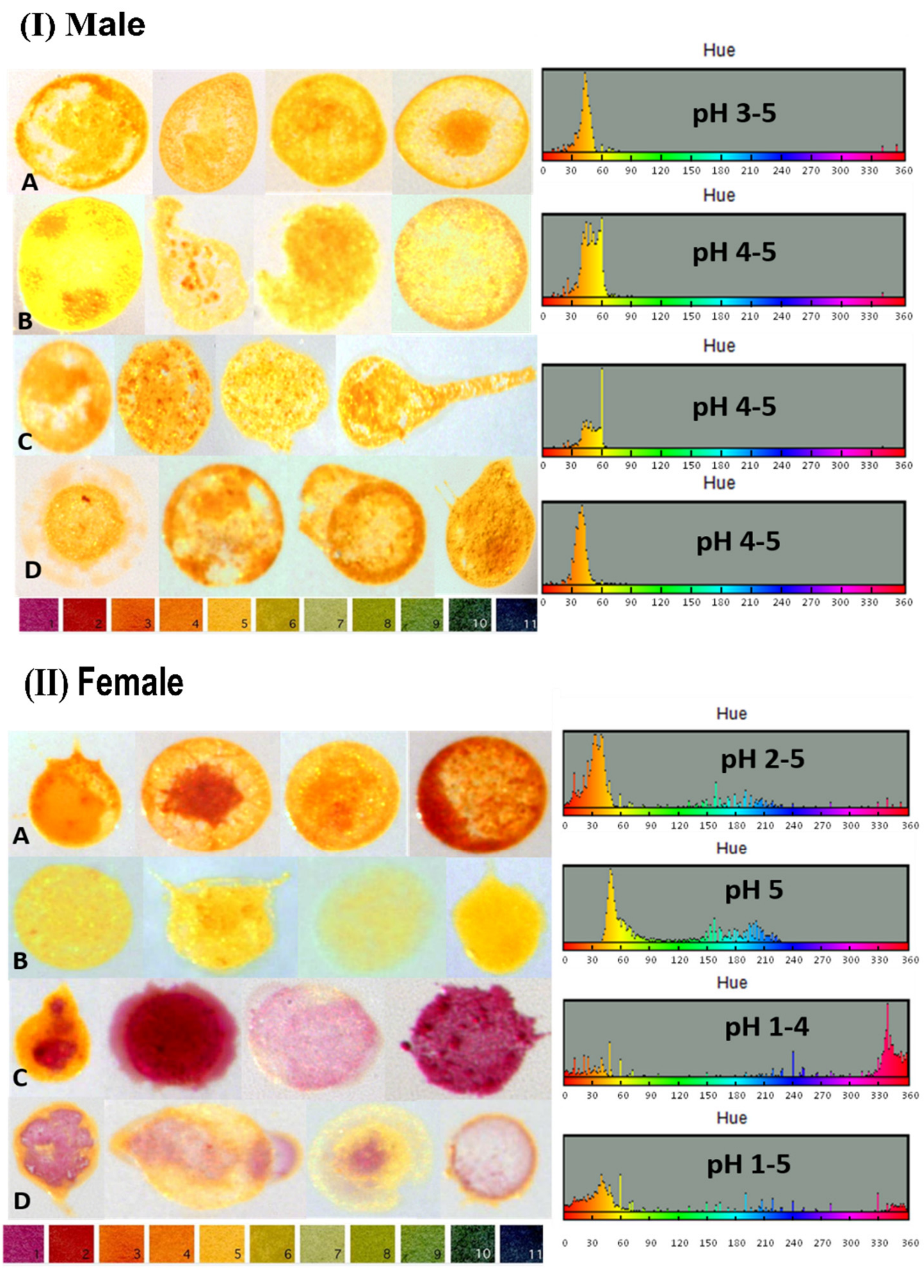


**Figure S2.** Hue, pH and patch color analysis of (I) male and (II) female (n = 6) fecal pellets after feeding on normal food media supplemented with different concentrations of dopamine. The regulation of pH is revealed by phenol red dye. A: No dopamine (control), B: 0.001 M, C: 0.0001 M and D: 0.00001 M dopamine in both males and females. The mean (Red-Green-Blue) converted to (Hue-Saturation-Brightness) and the corresponding color patch. The H value (hue) is the difference between the two lowest values divided by the maximum value and expressed in degrees (0–360). Hue values refer to the pH of the faecal pellets. Patch colors are based on hexadecimal HTML codes and are shown with their Hue value. A, females and B, males.

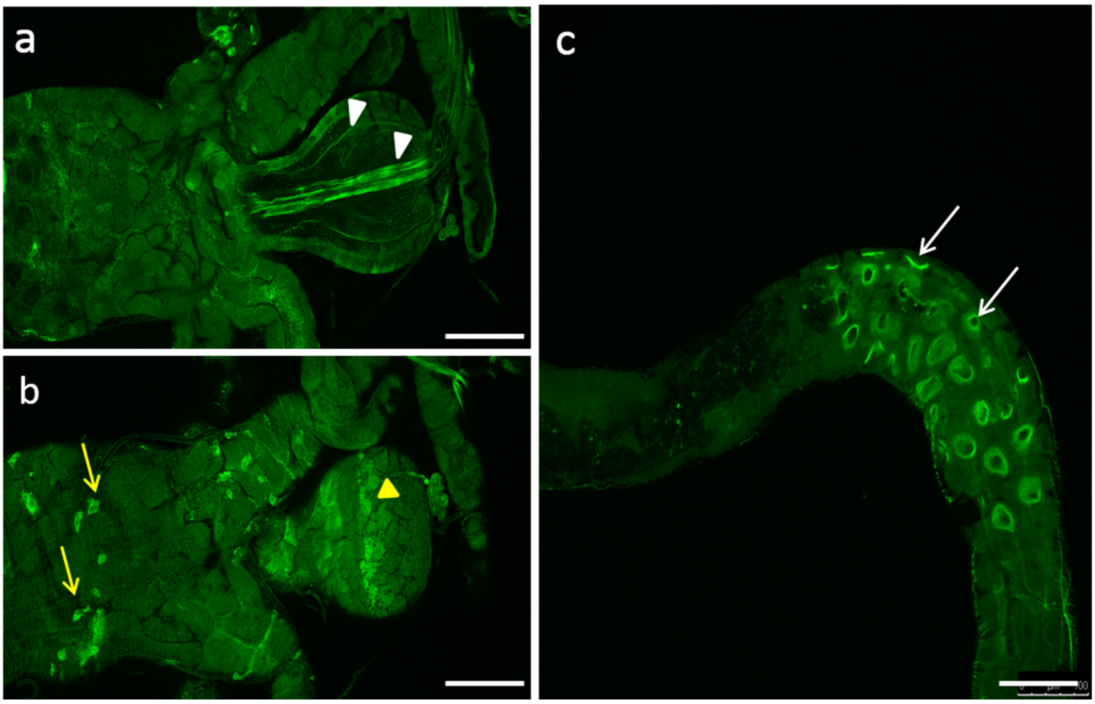


**Figure S3.** GFP immunoreactivity in the DoP1R1 GAL4::UAS GFP larval foregut a and b, and midgut, c. a and b are two confocal stacks for the same region. White head arrows refer to DoP1R1 in neurons innervating proventriculus region, Yellow head arrow refers to positive expression in proventriculus cells, Yellow arrows refer to DoP1R1 in enteroendocrine cells within the foregut, white arrows refer to the positive GFP signal in the copper cells.

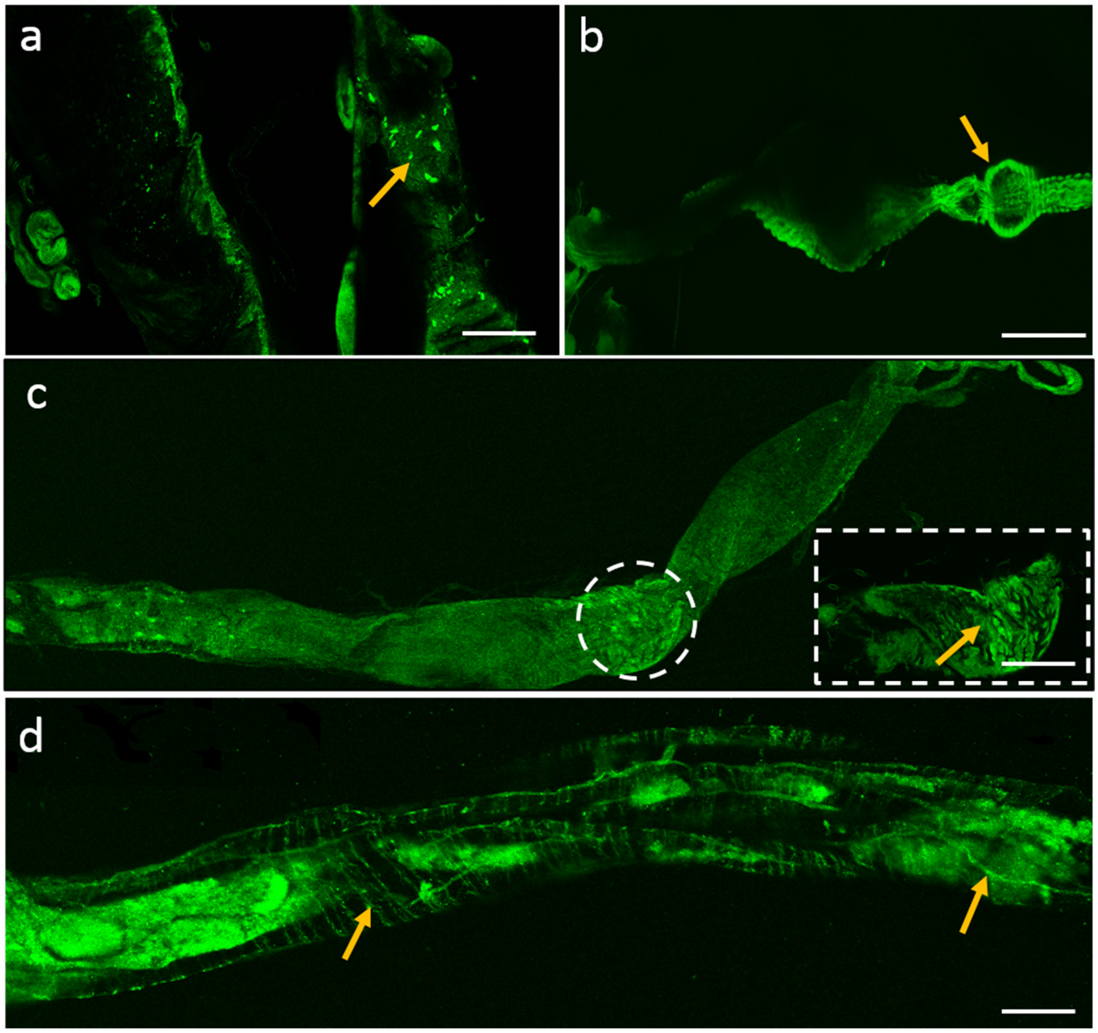


**Figure S4.** Dopamine receptors (DARs) in nerves and gut tissues of Adult *Drosophila* stained immunohistochemically using anti-GFP antibody. a, b: DoP1R1 mid gut tissues; c, d DoP1R2 in hind gut. Arrow in (**a**) refers to the presence of DoP1R1 in enteroendocrine cells. Arrow in (**b**) refers to the positive expression in the midgut enterocytes. (**c**) Dashed circle indicated the region with positive expression. Dashed rectangle in right side is the close view of this region, arrow refers to the positive expression of DoP1R2 in muscles of rectum (within ampulla). Arrows in (**d**) refer to the expression in neurons innervating midgut. Scale bars (**a**,**b**) 100 µm, (**c**) 75 µm, (**d**) 50 µm.

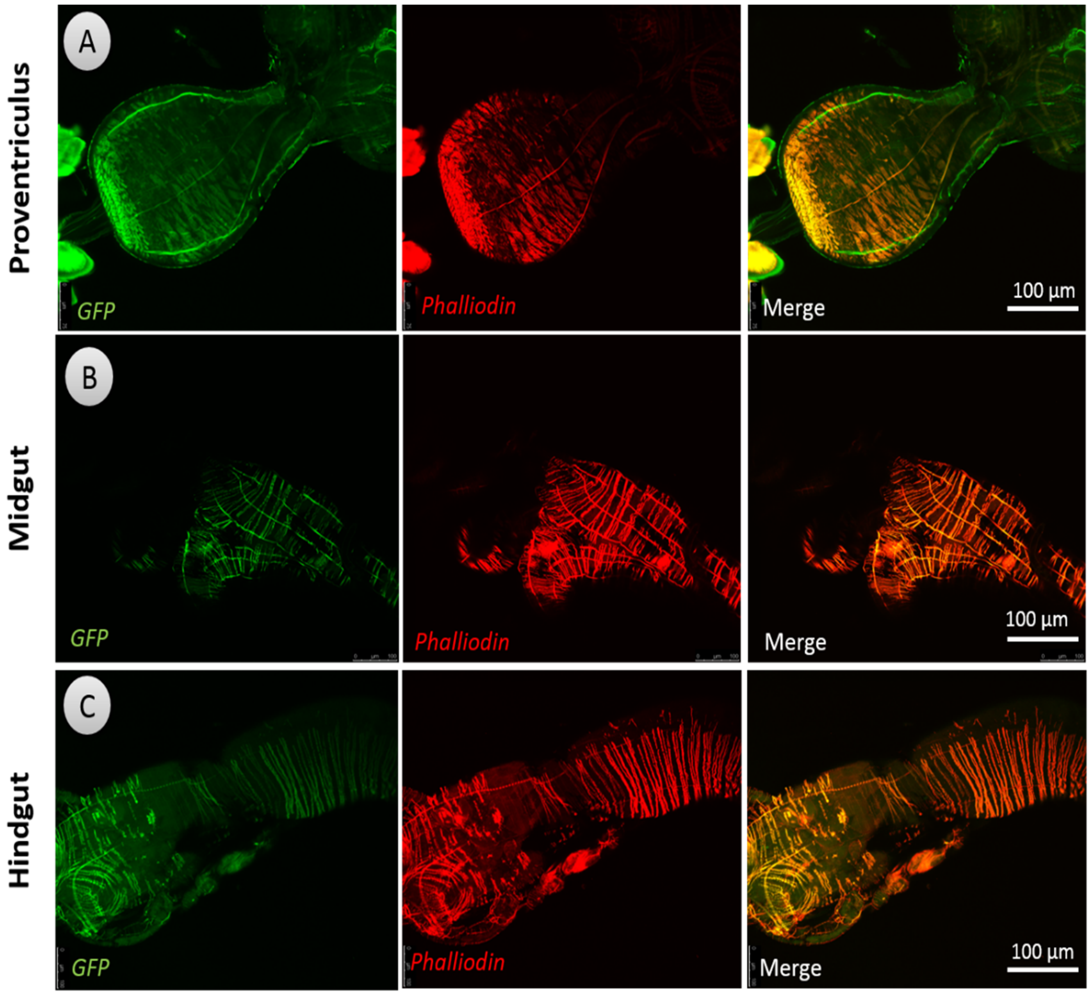


**Figure S5.** Dop1R2 expression pattern in larval gut musculature stained immunohistochemically using anti-GFP antibody (green florescent micrograph) with phalloidin (red florescent micrograph) and the overlay of these two florescent micrographs revealed that that almost all muscles express DoP1R2 receptor; (**A**) Proventriculus, (**B**) midgut and (**C**) hindgut. Note the GFP positive signal in the neurons innervating the proventriculus (did not stain with phalloidin).

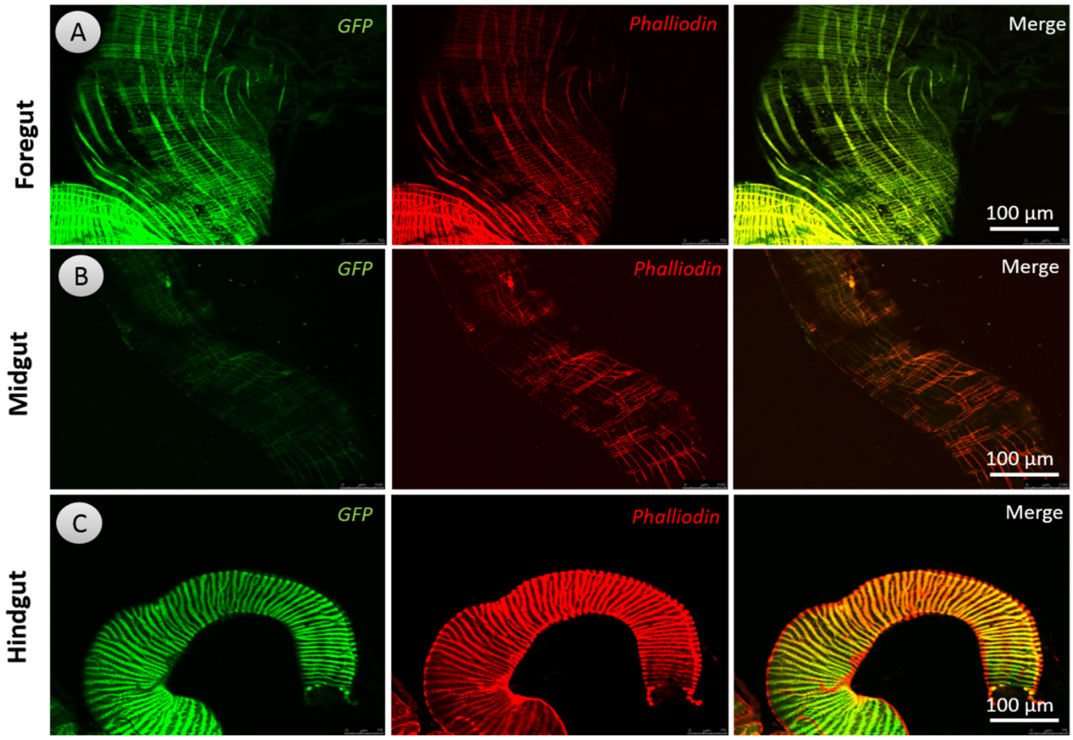


**Figure S6.** Dop1R2 expression pattern in Adult fly's gut musculature stained immunohistochemically using anti-GFP antibody (green florescent micrograph) with phalloidin (red florescent micrograph) and the overlay of these two florescent micrographs revealed that that almost all muscles express DoP1R2 receptor; (**A**) foregut, (**B**) midgut and (**C**) hindgut.

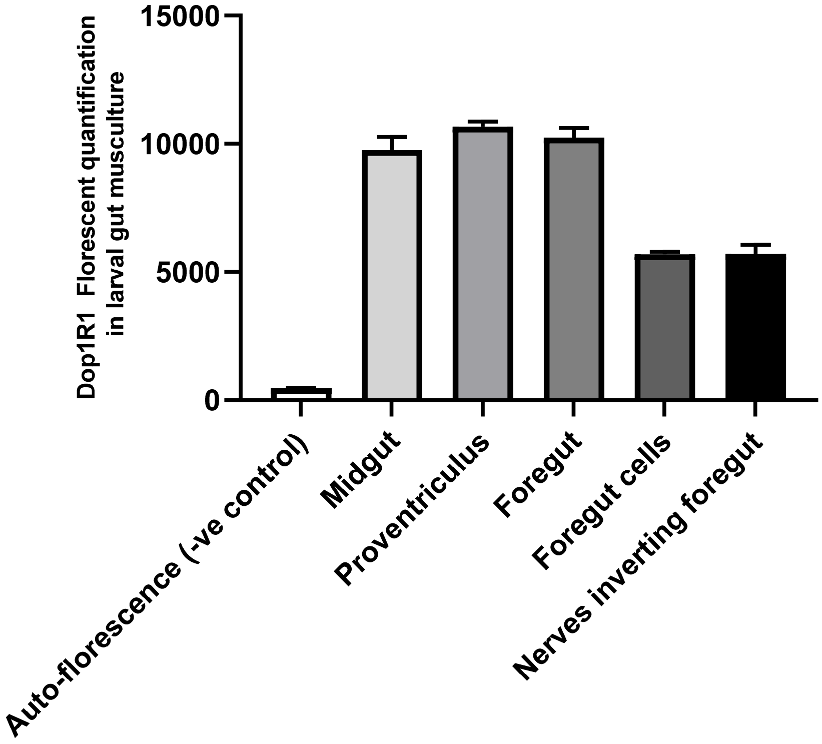


**Figure S7.** Quantification of Dop1R1 fluorescence localization in larval gut musculature. Dop2R data has been used as a negative control. The expression of Dop1R1 differed between gut tissues.

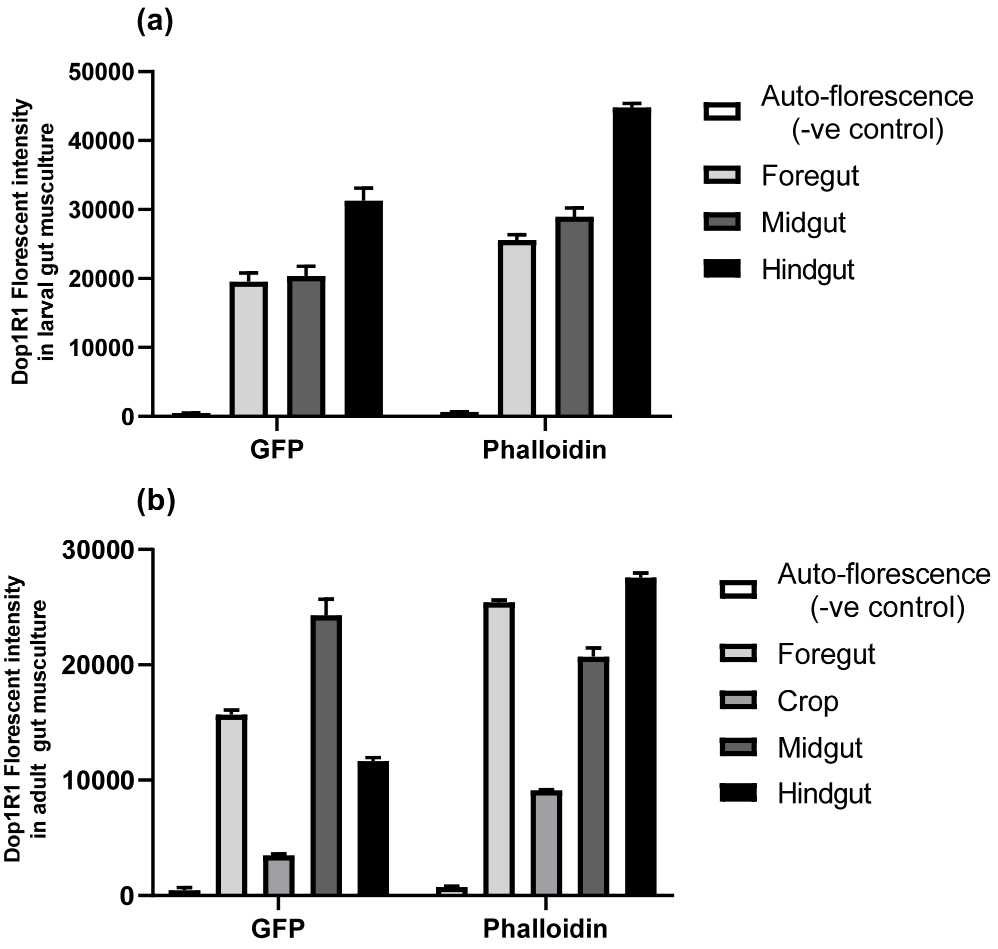


**Figure S8.** Quantification of Dop1R1 fluorescence localization in both larval and adult gut musculature. Dop2R data has been used as a negative control. The expression of Dop1R1 differed between gut tissues.

**Supplementary videos:**

**SV1.** Immunohistochemistry results showed that both Dop1R1 and Dop2R1 are expressed in Drosophila gut. To test whether the activation of those receptors via dopamine application will trigger the activation of PLC-β, crossing between special enterocytes GAL4 driver line namely, NP1-Gal4 with the effector line UAS PLC-β::mRFP was done. Guts of F1 adults of this crossing was dissected, then immediately 10-3M dopamine was applied on top of the gut. Results showed that dopamine trigger the translocation of PLC-β from the cytosol to the plasma membrane in comparison to the control. The PLC-β was then quickly returned back into cytosol after few seconds. This study showed using *ex vivo* assay that dopamine triggered the activity of PLC-β that leads to mobilization of intracellular Ca2+.
